# Ocoxin Increases the Antitumor Effect of BRAF Inhibition and Reduces Cancer Associated Fibroblast-Mediated Chemoresistance and Protumoral Activity in Metastatic Melanoma

**DOI:** 10.3390/nu13020686

**Published:** 2021-02-21

**Authors:** Aitor Benedicto, Iera Hernandez-Unzueta, Eduardo Sanz, Joana Márquez

**Affiliations:** 1 Department of Cell Biology and Histology, School of Medicine and Nursing, University of the Basque Country, 48940 Leioa, Bizkaia, Spain; aitor.benedicto@ehu.es (A.B.); ierajune@hotmail.com (I.H.-U.); 2 Research and Development, Catalysis S.L., 28016 Madrid, Spain; eduardo@catalysis.es

**Keywords:** melanoma, cancer nutrition, BRAF inhibition, fibroblasts, chemoresistance, adjuvant, tumor microenvironment

## Abstract

Whereas the prevalence of several cancer types is decreasing, skin malignancies are growing more common every year. Malignant melanoma is the most aggressive form of skin cancer with high metastatic capacity. In most cases, malignant melanoma shows acquired therapy resistance. We evaluated the ability of Ocoxin, a natural compound-based antioxidant and anti-inflammatory nutritional complement, to exert an antitumor effect in melanoma. To do so, the cytotoxicity of Ocoxin in a panel of BRAF-mutated murine and human melanoma cell lines was tested alone and in combination with BRAF inhibitor Vemurafenib. Our results revealed a potent cytotoxic effect of Ocoxin against melanoma cells and a synergic effect when combined with Vemurafenib, reducing viability and increasing apoptosis. Besides, Ocoxin interferes with the cell cycle, impairs the inherent and fibroblast-mediated melanoma cell migration, and reduces resistance to BRAF inhibition. Proteomic analysis revealed reduced tumor secretion of inflammatory factors Galectin-1, Osteopontin, CCL5, and CCL9 upon treatment with Ocoxin. Moreover, RNASeq showed that Ocoxin downregulated the cell cycle and proliferation-related genes. In vivo, Ocoxin reduced the number of lung metastasis of YUMM-1.7 melanoma cells. Therefore, Ocoxin arises as a good candidate for clinical trials analyzing the beneficial effects in patients suffering from this cutaneous malignancy.

## 1. Introduction

Cutaneous melanoma represents one of the most aggressive forms of melanoma and shows a highly metastatic phenotype. Despite the efforts to control and prevent this pathology, the incidence of this cancer is growing every year [1,2]. More than half of the newly diagnosed melanomas present the activating BRAF mutation BRAF V600E [1,2], which results in a dysregulated mitogen-activated protein kinase (MAPK) signaling activation. This mutation leads to uncontrolled proliferation and evasion of apoptosis, facilitating disease progression [1,3,4]. This mutation determines the treatment options for patients, with BRAF inhibitors (BRAFi) being the first-line targeted therapy. However, it is common for these patients to develop acquired resistance after a period of response. Therefore, there is an increasing need to find new approaches that may improve the antitumor effect of BRAFi while overcoming tumor resistance. Thus, combined therapy is being implemented for melanoma patients, targeting different pathways simultaneously, such as BRAF and MEK inhibition [5]. Moreover, due to the demonstrated antitumor effect of different phytochemicals such as green tea, curcumin, genistein, and resveratrol, its application as a coadjuvant therapy for cancer treatment has gained interest in recent years [6,7,8].

In this respect, Ocoxin is a mixture of several biological compounds, such as epigallocatechin-3-gallate, cinnamic acid, and vitamins B6, C, and B12, with demonstrated antitumor and immunomodulatory properties (Table 1). In this regard, Ocoxin reduces the tumor development of breast cancer, acute myeloid leukemia, liver metastasis of colorectal cancer, pancreatic cancer, and glioblastoma both in vitro and in vivo [9,10,11,12,13]. Moreover, clinical trials have revealed that Ocoxin was effective in increasing the quality of life and survival of patients receiving chemotherapy and radiotherapy while improving the tolerance to these treatments [14,15,16].

Ocoxin does not only exert a direct anticancer effect by increasing apoptosis and slowing down the cell cycle of tumor cells but also potentiates the cytotoxicity of several chemotherapeutic agents [9,10,11,12,13,17]. This synergistic effect may improve the action of anticancer drugs that lose effectiveness in vivo due to cancer cell intrinsic and acquired resistance. In this last process, the tumor microenvironment (TME) arises as the main player, where tumor cell/stromal cell interactions lead to treatment resistance [18,19]. The TME is composed by cancer cells, cancer-associated fibroblasts (CAFs), tumor-associated macrophages, recruited immune populations, the extracellular matrix (ECM), and growth factors, cytokines, and extracellular vesicles secreted by different cell types [20]. CAFs represent the main cell type in the TME and are under the spotlight due to their ability to promote tumorigenesis by means of chemoresistance, increased tumor cell proliferation, migration, and ECM remodeling. In fact, CAFs have been proposed as a target for anticancer therapies [21,22,23]. Besides, previous studies of this group revealed that Ocoxin reduced the migratory capacity of CAFs and the infiltration of hepatic stellate cells (HSCs) into the liver metastatic foci of colorectal cancer (CRC) in vivo [10]. HSCs are liver resident cells that become myofibroblast-like cells upon activation, and their relations with cancer progression are widely recognized [24]. Moreover, Ocoxin reduced the chemoresistance induced by fibroblasts-derived secretomes in pancreatic cancer cells [9].

Thus, due to the wide spectrum of antitumor capacities demonstrated by Ocoxin, we aimed to evaluate the antitumor potential of this compound in the progression of metastatic melanoma, its possible synergic effect with the BRAF inhibitor Vemurafenib, and its ability to modulate melanoma-CAF crosstalk driving to chemoresistance and disease progression in vitro and in vivo.

## 2. Materials and Methods

### 2.1. Cell Lines

A panel of 4 melanoma cell lines was analyzed in this study. The novel BRAF mutated mouse metastatic melanoma cell line YUMM-1.7 (ATCC, Manassas, VA, USA) was grown in RPMI-1640 medium (Life Technologies, Carlsbad, CA, USA). Human metastatic melanoma cell lines HT-144, RPMI-7659 (ATCC, Manassas, VA, USA), and COLO-800 (Sigma-Aldrich, St. Louis, MO, USA) were cultured in, McCoy´s, DMEM, and RPMI-1640 mediums, respectively. Murine 3T3 and human lung MRC-5 fibroblasts (ATCC, Manassas, USA) were cultured in DMEM and RPMI-1640, respectively. All cell lines were supplemented with 10% FBS, penicillin (100 U/mL), streptomycin (100 μg/mL), and amphotericin B (0.25 μg/mL) (ThermoFisher Scientific, MA, USA). Cells were cultured at 37 °C in a humidified atmosphere in the presence of 5% CO_2_.

### 2.2. Animals

We obtained 6–8-week-old male mice from C57BL/6 background from Charles Rivers (Barcelona, Spain). Mice maintained in line with institutional guidelines and national and international laws for experimental animal care. The experimental procedures were approved by the Basque Country University Ethical Committee (CEID) and by institutional, national, and international guidelines regarding the protection and care of animals used for scientific purposes (Reference M20-2018-114).

### 2.3. Cell Viability Assay

For cell viability assays, 5 × 10^3^ tumor cells were cultured in 96-well plates in complete medium for 18 h. Then, melanoma cells were treated with several dilutions of Ocoxin (Catalysis S.L., Madrid, Spain) ranging from 1:1000 to 1:50 (V/fV) in 1% FBS supplemented medium. For BRAF inhibition, cells were treated with 0.1, 1 and 10 μM Vemurafenib (Euroasian Chemicals, Mumbai, India) in 1% FBS supplemented medium. Finally, to analyze the potential of Ocoxin as a coadjuvant therapy for BRAF inhibition, cells were treated with Ocoxin (1:50) in combination with different concentrations of Vemurafenib. Control cells were cultured with 1% FBS supplemented medium containing antibiotics, and antimycotics. Cell viability was measured after 24 and 48 h using Prestoblue Viability Reagent (Thermo Fisher Scientific, MA, USA) following manufacturer`s indications. Briefly, cells were incubated with Prestoblue Viability reagent diluted 1:10 in fresh medium for 2 h. To obtain cell viability percentage, the obtained absorbance was converted to percentage of control and compared to treatment concentration.

### 2.4. Cell Cycle Analysis

For cell cycle analysis, 3 × 10^5^ melanoma cells were cultured in 6-well plates for 18 h in complete medium. Cells were treated with 1:50 dilution of Ocoxin for 48 h in 1% FBS supplemented medium. Control cells were cultured with 1% FBS supplemented fresh medium without Ocoxin. Cells were then trypsinized, washed once with phosphate-buffered saline (PBS), and fixed with 70% ethanol for 30 min at 4 °C. Afterward, cells were washed with PBS twice and incubated with propidium iodide (PI) containing FxCycle PI/RNase Solution (Thermo Fisher Scientific, MA, USA) following the manufacturer’s indications. Finally, changes in the cell cycle were studied by flow cytometry using the Gallios cytometer (Beckman Coulter, Brea, CA, USA).

### 2.5. Apoptotic Cell Detection

3 × 10^5^ melanoma cells/well were cultured in complete medium for 18 h in 6-well plates. The medium was replaced by fresh medium supplemented with 1% FBS for the control condition or treated with Ocoxin 1:50, Vemurafenib 1 µM or the combination of both treatments for 48 h. Then, cells were washed with PBS, trypsinized, pelleted by centrifugation, washed 2 times with PBS, and double stained with the annexin V/IP apoptosis detection kit following manufacturer’s instructions (Thermo Fisher Scientific). Finally, apoptosis was determined by the Gallios cytometer (Beckman Coulter).

### 2.6. Generation of Tumor Cell and Tumor-Stimulated Fibroblast-Derived Secretomes

Tumor-derived secretomes were obtained after culturing 2 × 10^5^ cells/mL in 24 well plates for 18 h in 10% FBS supplemented RPMI medium. Then, that medium was changed for serum free medium and 24 h later secretomes were collected. Tumor-stimulated fibroblasts derived secretomes (TS-fibroblast secretomes) were obtained from murine 3T3 and human MRC-5 cell lines. Briefly, cells were cultured on 24-well plates at a concentration of 2 × 10^5^ cells/mL in 10% FBS supplemented RPMI medium. After 18 h, the medium was replaced for tumor-derived secretomes diluted 1:2 in fresh serum-free medium and incubated for another 24 h. Afterward, the medium was changed for serum-free RPMI medium and incubated for 24 h. All the obtained secretomes were collected, centrifuged for 5 min at 4000 rpm and stored at −20 °C.

### 2.7. Transwell Migration Assay

The melanoma cell migration assay was carried out using Modified Boyden chambers. First, 1 × 10^4^ cancer cells were cultured onto 8 μm-diameter pore membranes (Greiner Bio-one, Kremsmünster, Austria) and allowed to adhere and spread for 3 h before the addition of different treatments. Then, the medium was changed without disturbing the top layer of the insert for fresh medium or TS-fibroblast secretomes diluted 1:2 in fresh medium supplemented with 1% FBS with or without Ocoxin diluted 1:50. After 20 h, the migrated cell numbers were quantified in the insert membranes by means of 4% formalin fixation, Cristal Violet staining (0.4%) (Sigma-Aldrich, St. Louis, MO, USA) or DAPI staining (COLO-800 and HT-144) and mounted for the quantification under the microscope. Ten fields using 200× magnification were counted per membrane. Data are expressed relative to the migration of untreated melanoma cells.

### 2.8. YUMM-1.7 Melanoma Cell Secretome Analysis

The YUMM-1.7 cell secretomes were obtained as previously described. Briefly, 2 × 10^5^ cells/mL were cultured in 24-well plates for 18 h in complete medium. Then, YUMM-1.7 cells were treated with 1:50 Ocoxin dilution for 24 h, followed by incubation with serum free medium for another 24 h. Finally, the medium rich in YUMM-1.7 secreted mediators was collected and centrifuged for 5 min to 4000 rpm and stored at −20 °C. The secretome of YUMM-1.7 was analyzed using Mouse Cytokine Antibody Array Membrane kit (Abcam, UK) following manufacturer’s instructions. The obtained spot intensities were normalized to positive controls and relativized regarding Ocoxin treated cell viability vs. Control cell viability.

### 2.9. RNASeq for COLO-800 Melanoma Cell Gene Expression Analysis

To study the changes promoted by Ocoxin in human melanoma cells, 3 × 10^5^ COLO 800 cells were cultured in 6-well plates in 10% FBS for 18 h. Afterward, cells were treated with 1:50 Ocoxin dilution in 1% FBS supplemented medium for 48 h. Finally, dead cells were discarded through PBS washing and the RNA of adhered cells was isolated. Three sample replicates were analyzed for each treatment.

### 2.10. Transcriptomic Analysis of Human Samples

The analysis of human transcripts in skin cutaneous melanoma (SKMC) (n = 461) and normal tissue (558) was carried out through the GEPIA Database (http://gepia.cancer-pku.cn (accessed on 12 December 2020)). The expression of Galectin-1 (LGALS1), Osteopontin (OPN), CCL5 and CCL15 (the human homolog of mouse CCL9) was compared between SKCM and normal tissue using boxplot analysis (Expression DIY, Log2FC| Cutoff: 1, *p*-value cutoff: 0.01, Log scale and Jitter Size 0.4). TCGA normal and GTEx data were selected as matched normal data. Data are expressed as transcripts per million (TPM).

### 2.11. In Vivo Lung Metastasis Procedure

For the in vivo development of lung metastasis, 1 × 10^6^ YUMM-1.7 cells diluted in PBS (100 µL) were injected through the tail vein of C57BL/6 mice, and the animals were randomly divided in 4 groups (Figure S1). As the development of lung metastasis is slow in this model [25], treatments started 4 months after tumor cell inoculation. Control mice were treated with vehicle solution (4% DMSO, 5% Tween-100, 30% Polyetilenglycol, 61% distilled water). The second group was treated with daily oral administration of Ocoxin (100 µL). The third group received intraperitoneal injections of Vemurafenib every other day, 3 days a week (50 mg/Kg diluted in 4% DMSO, 5% Tween-100, 30% Polyetilenglycol and 61% distilled water). The combination group received the combination of both treatments. The Control group and Ocoxin group received intraperitoneal injections of vehicle solution every other day, 3 days a week. After 30 days of treatment, mice were sacrificed, and lungs were fixed for paraffin histological analyses.

### 2.12. Immunohistochemical Analysis

In order to analyze the tumor development on the lung, we performed an immunohistochemical staining for microphthalmia-associated transcription factor (MITF), which is expressed in YUMM 1.7 melanoma cells (Figure S2). To do so, first, antigen retrieval was carried out in citrate buffer pH 6.0. Then, endogenous peroxidase and nonspecific proteins were blocked by incubating the samples for 40 min with 3% of H_2_O_2_ and 40 min with 3% FBS. Then, lung tissue slides were incubated overnight with a specific MIFT antibody (1:500 Thermo Fisher Scientific; Waltham, MA, USA). Next, sections were washed, and the secondary antibody was added. Finally, the antigen expression was revealed by a horseradish peroxidase (HRP)-conjugated streptavidin (Life Technologies) and 2-Solution DAB kit (Life Technologies) following the manufacturer’s instructions. Antigen expression levels were quantified by ImageJ software (NIH, Bethesda, MD, USA). Results were expressed as the mean percentage MITF positive area for each group (n = 12).

### 2.13. Statistical Analysis

The statistical analysis was carried out using the Student’s 2-tailed unpaired *t*-test. All the in vitro experiments were performed in triplicate, and the in vivo assay was carried in duplicate with at least 6 animals in each group for each replicate. Data are expressed as the mean value (+/− standard deviation [SD]). The RNASeq assay was performed with 3 replicates for each treatment, and the statistics were analyzed with the multiExperiment Viewer version 4.9.0 (http://www.tm4.org/mev/ (accessed on 24 October 2019)).

## 3. Results

### 3.1. Cytotoxic Effect of Ocoxin in Human and Murine Melanoma Cell Lines

**Figure 1:**
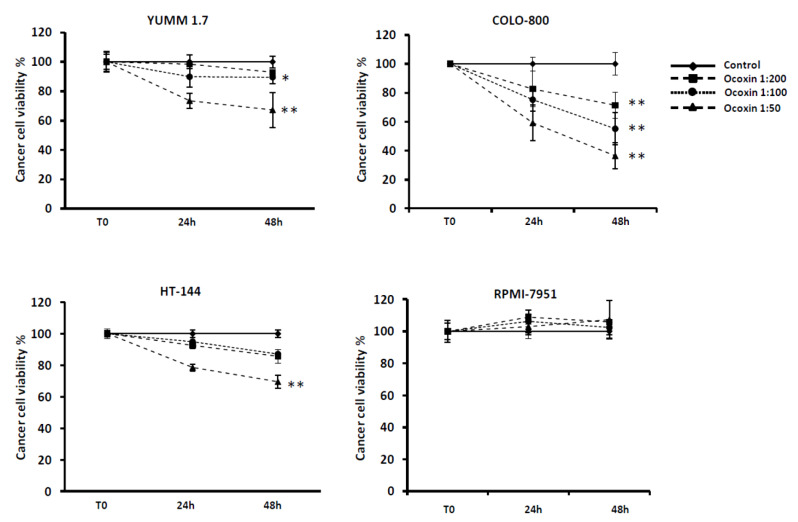
Cytotoxic effect of Ocoxin in a melanoma cell panel in vitro. The potential of Ocoxin to reduce tumor cell viability was assessed in vitro. To do so, four melanoma cell lines were treated with different concentrations of Ocoxin for 24 h and 48 h, and cell viability was analyzed through PrestoBlue Cell Viability Assay. We reported a dose-dependent effect of the compound in three out of four melanoma tested cell lines. Data are represented as the percentage of viable cells compared to that of untreated cells. The results show the mean of three different experiments ± SD. Statistical differences are represented as *: *p* < 0.05; **: *p* < 0.01 by one-way ANOVA test.

The antitumor potential of Ocoxin was studied in foru BRAF-mutated melanoma cell lines, including the murine YUMM-1.7 and the human COLO-800, HT-144, and RPMI-7951. All the tumor cells were exposed for 24 h and 48 h to different dilutions of the natural compound Ocoxin. After these incubation periods, cell viability was measured. As shown in Figure 1, Ocoxin exerted an antitumor effect in three out of four melanoma cell lines by means of reduced cell viability in a dose-dependent manner. The 1:50 dilution showed the highest cytotoxic effect, ranging from 30% decreased viability in YUMM 1.7 and HT-144 to 60% in COLO-800. However, no effect was observed in RPMI-7951 cells after the exposure to Ocoxin.

### 3.2. The Antitumor Effect of Ocoxin is Mediated by Apoptosis and Cell Cycle Arrest in Melanoma Cells

**Figure 2:**
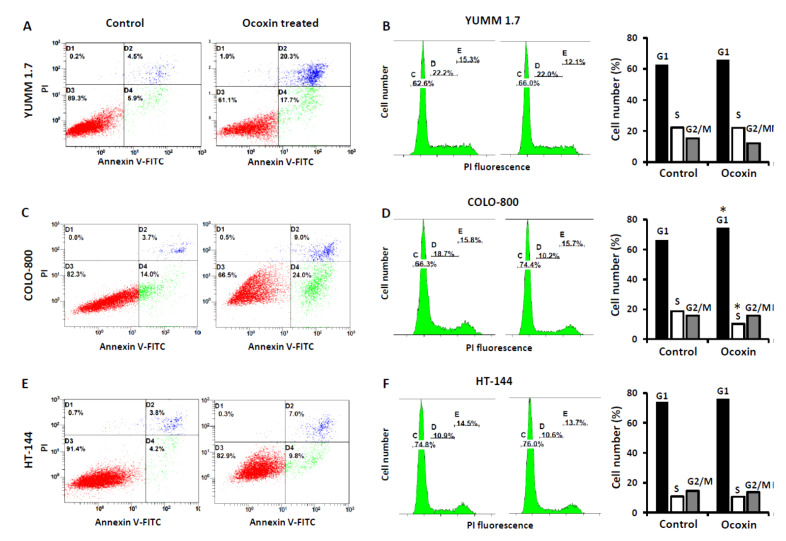
Mechanism of action of Ocoxin in vitro. Responding melanoma cells were analyzed for apoptosis and cell cycle regulation upon Ocoxin treatment. Cells were treated for 48 h with the 1:50 dilution of Ocoxin. (**A**,**C**,**E**) Cells were incubated with the Anexin V/PI Apoptosis Kit for the quantification of apoptotic cell number. (**B**,**D**,**F**) On the other hand, cells were incubated with propidium iodide (PI) and the cell cycle was studied. The experiments were carried out at least two times. The results show a representative experiment. *: *p* < 0.05; by unpaired *t*-test.

In order to uncover Ocoxin-mediated tumor cell viability reduction, apoptosis and cell cycle arrest were analyzed. Interestingly, the viability decrease seems to be partly mediated by apoptotic cell death as observed through Anexin V/PI assay in cancer cells incubated for 48 h with Ocoxin 1:50 dilution (Figure 2). Ocoxin increased apoptotic cell counts in three out of four cells studied. YUMM 1.7 cell apoptosis increased three-fold upon Ocoxin treatment, while COLO-800 and HT-144 apoptotic cell counts increased two-fold (Figure 2A,C,E). To evaluate the ability of this compound to interfere with cancer cell cycle, tumor cells were incubated for 48 h with 1:50 dilution. Afterward, the cell cycle was analyzed using the FxCycle™ PI/RNase Staining Solution. The treatment with Ocoxin drove the accumulation of tumor cells in the G0/G1 phase and decreased the S phase cell number in COLO-800 melanoma cells and slightly in YUMMM-1.7 cells. However, the HT-144 cell cycle was not affected upon Ocoxin treatment. In detail, the G0/G1 population increased from 63.05% to 66.5% in YUMM-1.7 cells, 66.3% to 74.4% in COLO-800 cells, and 74.1% to 76% in HT-144 melanoma cells (Figure 2B,D,F).

### 3.3. Vemurafenib Mediated Cell Death Increases in Combination with Ocoxin Treatment

**Figure 3:**
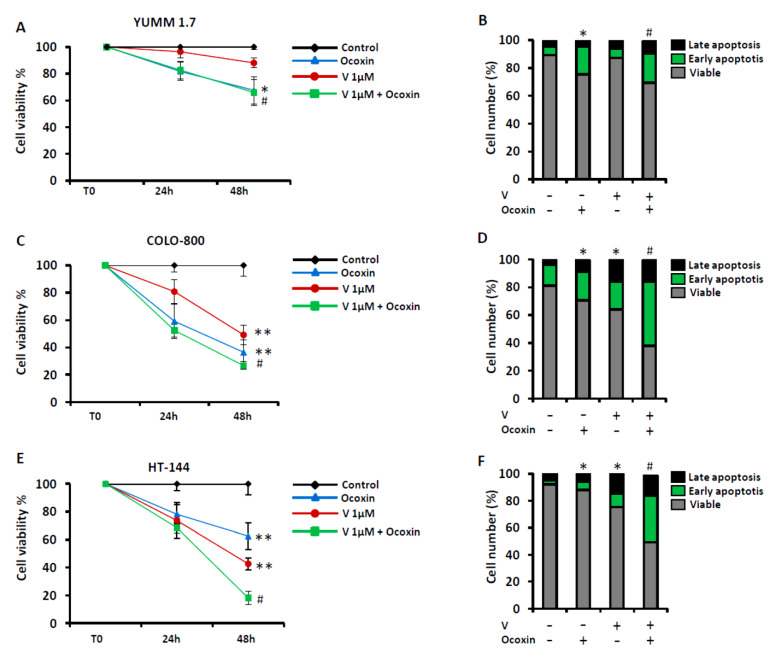
Ocoxin increased the antitumor effect of BRAF inhibition in vitro. (**A**,**C**,**E**) Melanoma cells were treated with BRAF inhibitor Vemurafenib (1 µM) alone or in combination with Ocoxin 1:50 concentration for 24 h and 48 h and cell viability was measured by the Prestoblue viability assay. (**B**,**D**,**F**) Melanoma cells were treated with the same combination regimens for 48 h and viable cells, early and late apoptosis were measured though Anexin V/PI apoptosis Kit. Experiments were carried out three times and results show the mean of these replicates ± SD. Statistical differences are represented as * *p* < 0.05 and ** *p* < 0.01 between untreated cells and Ocoxin- or Vemurafenib-treated cells by one-way ANOVA test. ^#^ *p* < 0.05 between Vemurafenib treatment alone and Vemurafenib and Ocoxin combination treatment.

The inhibition of BRAF is the main strategy to treat melanoma patients with BRAF mutation. Interestingly, the effect of Vemurafenib, a BRAF inhibitor, was significantly increased in combination with Ocoxin in vitro. Melanoma cell viability was reduced after 24 h and 48 h when treated with the combination of Vemurafenib 1 µM and 1:50 dilution of Ocoxin compared to the single treatment viability (Figure 3A,C,E). Moreover, BRAF inhibition-mediated apoptotic cell death was increased when combined with Ocoxin. Treatment of YUMM-1.7 with 1 μM led to an 87.2% of viable cells, along with an 11.5% apoptotic cells. However, the combination of 1 μM Vemurafenib with the 1:50 dilution of Ocoxin reduced the viable cell counts down to 69.4% with 29.8% of apoptotic cells (Figure 3B). In this case, Ocoxin alone showed a similar effect as the combined treatment, as observed in Figure 3B with overlapped lines. Vemurafenib 1 µM treatment resulted in 64% viability of COLO-800 with 20.3% of apoptotic cells, while cotreatment with Ocoxin reduced viability down to 37.9%, increasing apoptosis up to 46.3% (Figure 3D). The same effect was reported in the HT-144 cell line upon Ocoxin cotreatment, with a 20% reduction in cell viability and 2.5-fold apoptosis increase compared to that of Vemurafenib alone (Figure 3E,F) (Figures S3–S5).

### 3.4. Fibroblast-Mediated Chemoresistance to Vemurafenib Is Partially Reverted by Ocoxin

**Figure 4:**
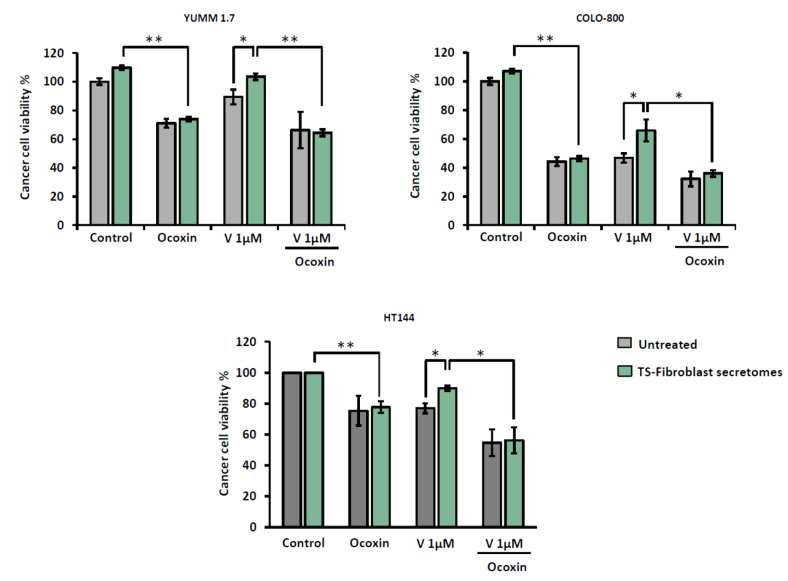
Ocoxin impairs TS-fibroblast-mediated resistance to BRAF inhibition in vitro. Melanoma cells were treated with fresh medium or TS-fibroblast-derived secretomes for 24 h. Afterward, cells were treated with BRAF inhibitor Vemurafenib (1 µM) alone or in combination with Ocoxin 1:50 concentration diluted in fresh medium or TS-fibroblast-derived secretomes for 48 h and cell viability was measured. The results show the mean of three independent experiments ± SD. Statistical differences are represented as * *p* < 0.05; ** *p* < 0.01 by one-way ANOVA test.

The role of TS-fibroblasts during tumor progression involves increased chemoresistance of cancer cells to different anticancer drugs [26,27]. It has been shown that TS-fibroblasts mediate resistance upon BRAF inhibition in melanoma [28]. Here, we show that TS-fibroblasts-derived secretomes reduced the cytotoxic effect of Vemurafenib 1 µM in melanoma cells (Figure 4). TS-fibroblast secretomes diminished Vemurafenib cytotoxicity in YUMM 1.7 cells and partially abrogated antitumor effect of BRAF inhibition in COLO-800 and HT144 cells. Interestingly, Ocoxin cotreatment with Vemurafenib partially overcame TS-fibroblast-mediated resistance in melanoma cells, boosting the anticancer activity of BRAF inhibition (Figure 4).

### 3.5. Promigratory Effect of TS-Fibroblasts on Tumor Cells Is Diminished by Ocoxin

**Figure 5:**
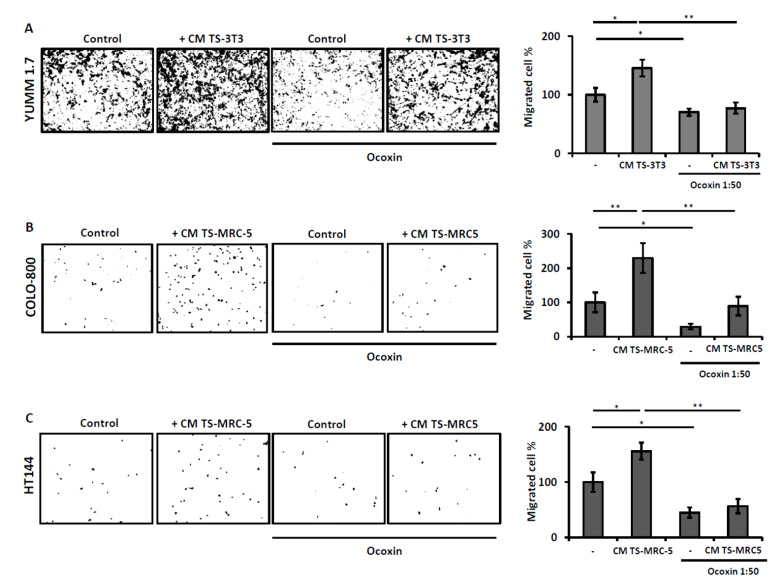
Ocoxin reduced TS-fibroblast secretomes-mediated melanoma migration in vitro. Melanoma cells were tested for migration upon treatment with TS-fibroblast secretomes and Ocoxin. Melanoma cells were treated with fresh medium or TS-fibroblast-derived secretomes supplemented with 1:50 dilution of Ocoxin for 20 h. Afterward, inserts were fixed and stained for the quantification under light and fluorescence microscope. (**A**) YUMM 1.7 cells (**B**) Colo 800 cells (**C**) HT 144 cells. The results show the mean of three independent experiments ± SD. Representative images for one 200× field for each treatment are shown. Statistical differences are represented as * *p* < 0.05 and ** *p* < 0.01 by one-way ANOVA test.

Tumor migration is a critical step during metastasis and tumor progression. Fibroblasts are one of the main components of the tumor microenvironment and promote different protumorigenic processes. We found that TS-fibroblast-derived secretomes stimulate the migration of all melanoma cells analyzed after 20 h. In fact, TS-fibroblasts secretomes enhanced the migration of YUMM-1.7 up to 50% compared to untreated tumor cells (Figure 5A). The same trend was observed in human cells lines, with 100% increased migratory potential in COLO-800 and 50% in HT144 cells (Figure 5B,C). Interestingly, tumor cell treatment with Ocoxin led to 30% reduced migration in YUMM 1.7, 60% in COLO-800, and 50% in HT144 melanoma cells. Moreover, Ocoxin reverted the promigratory and stimulatory effect of TS-fibroblast secretomes in tumor cells, reducing tumor cell migration to basal levels (Figure 5). It is to note that this reported reduced migration may be also related to Ocoxin cytotoxicity and not only to impaired migratory effect of this compound in melanoma cells.

### 3.6. Ocoxin Alters the Secretome of YUMM 1.7 Melanoma Cells

**Figure 6:**
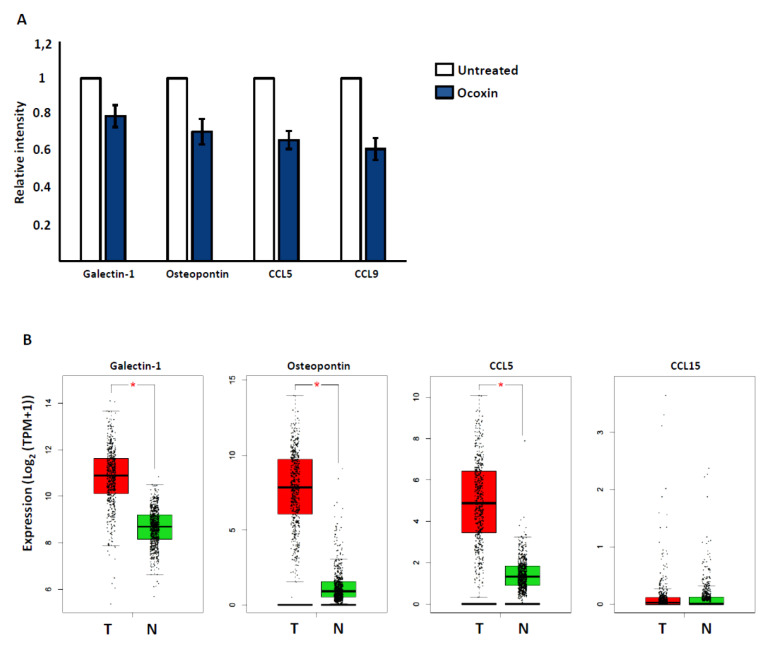
Coxin treatment modulated the secretome of YUMM 1.7 cells in vitro. We analyzed the changes in a wide array of secreted cytokines and growth factors upon Ocoxin stimulation in YUMM 1.7 cells. (**A**) YUMM 1.7 cells were treated for 24 h with Ocoxin. Afterward, the medium was changed for fresh medium. After 24 h, the secretome was collected, analyzed through cytokine array assay, and compared to that of untreated cells. The results show the mean of two replicates ± SD. Representative images for cytokine array membranes are shown. (**B**) The gene expression of the proteins downregulated by Ocoxin were analyzed in melanoma patients and compared to healthy tissue. Statistically significant differences are represented as * *p* < 0.05 by one-way ANOVA test.

The creation of a favorable tumor microenvironment is a prerequisite for successful metastatic growth. Soluble factors secreted by tumor cells mediate the recruitment and activation of nontumoral cells. We found that Ocoxin treatment reduced the secretion of several proinflammatory mediators by melanoma cells. In detail, Ocoxin suppressed the secretion of LGALS1, OPN, CCL5, and CCL9 up to 20%, 25%, 37%, and 40%, respectively (Figure 6A) (Figure S6). Interestingly, we found that the expression of LGALS1, OPN, and CCL5 was significantly overexpressed in cancerous tissue (T) compared to normal samples (N) in patients suffering from skin melanoma (Figure 6B), while no differences were found for CCL15, the homolog of mouse CCL9 in humans.

### 3.7. RNASeq Analysis of Human COLO-800 Melanoma Cells

**Figure 7:**
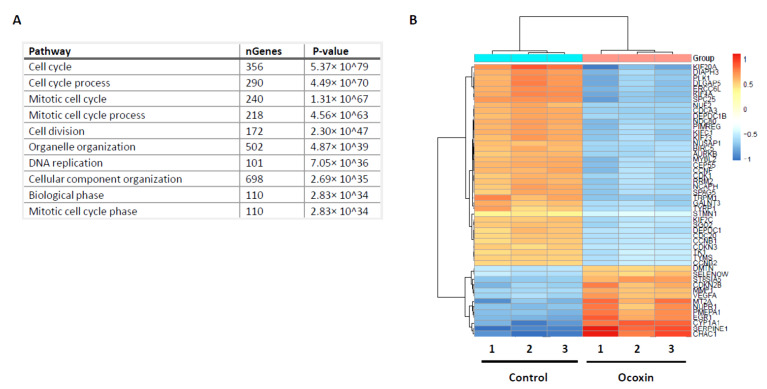
Gene expression changes in COLO-800 human melanoma cell line. COLO-800 melanoma cell line was treated with Ocoxin for 48 h. Changes in RNA levels were measured through RNASeq in control and Ocoxin-treated COLO-800. (**A**) Most significantly downregulated pathways. (**B**) Heatmap of the most significant gene changes in RNASeq of untreated vs. Ocoxin-treated cells.

The effect of Ocoxin was studied by means of genetic alterations of the expression patterns of human melanoma COLO-800 cells. Treatment of melanoma cells with Ocoxin 1:50 dilution for 48 h upregulated the expression of 1406 genes, while Ocoxin treatment downregulated the expression of 1673 genes. Interestingly, most of the genes silenced by Ocoxin were related to proliferation and the cell cycle (Figure 7A). As observed in Figure 7A, 7 out of 10 most significantly downregulated GO pathways were directly related to the cell cycle and mitosis. In fact, the heatmap for altered genes revealed that Ocoxin altered the expression of several genes directly related to malignant phenotype of cancer cells (Figure 7B).

### 3.8. Ocoxin Administration Reduces Lung Metastasis In Vivo

**Figure 8:**
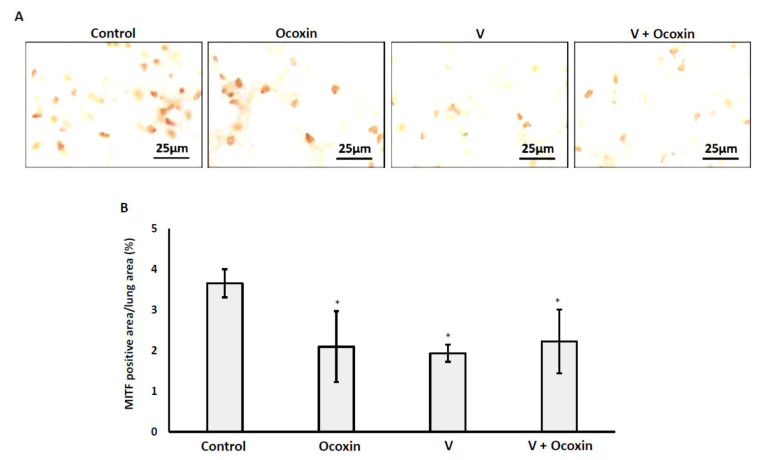
Effect of Ocoxin in an experimental model of melanoma lung metastasis. Mice were intravenously injected with 1 × 10^6^ YUMM 1.7 melanoma cells. After 4 months, mice were treated with different regimens for 30 days. Afterward, lungs were collected and fixed for their analysis. (**A**) A representative image of MITF expression in lungs of each group is shown. (**B**) The mean area occupied by MITF positive YUMM 1.7 cells under different treatment regimens is represented. Statistically significant differences are represented as * *p* < 0.05 by unpaired *t*-test.

The metastatic spread of the primary lesion is the worst scenario when dealing with melanoma. In fact, melanoma is the most aggressive skin malignancy, giving rise to lung and brain metastasis in most of the patients suffering from distant lesions [29,30]. To study the antitumor effect of Ocoxin, we analyzed the lung metastasis of YUMM-1.7 melanoma upon different treatment routines. We observed decreased metastatic burden in mice treated with Ocoxin and Vemurafenib compared to vehicle-treated mice (Figure 8A,B). The combination of both treatments showed a reduction trend but did not show a significant relation compared to both treatments alone. It should be noted that the overall state of the mice receiving the combination regimen was better than that of mice receiving Vemurafenib alone. We found that a low number of mice exhibited metastatic cells in the lung after intravenous injection of YUMM 1.7 (Figure S7), which is in line with previous studies [25].

## 4. Discussion

Cutaneous melanoma is one of the most aggressive cancers due to its high capacity to disseminate. However, due to the increasing knowledge of the molecular and cellular mechanism underlying this malignancy, several treatment options, such as immunotherapy and targeted therapy, have been developed in recent years with improved clinical outcomes. BRAF and MEK inhibitors are the first-line treatment when the activated BRAF mutation is detected in melanoma patients [31,32]. Nevertheless, the resistance to these treatments produced by intrinsic tumor mechanisms or external factors is a common adverse scenario in several cases [33,34,35]. Thus, the new alternatives to cope with this malignancy are imperative. Ocoxin, a mixture of several biological compounds such as green tea polyphenols, ascorbic acid, and vitamins, has demonstrated antioxidant, anti-inflammatory, and antitumoral properties in several cancer types [9,10,11,12,13,17,36]. In this regard, previous studies demonstrated that antioxidants inhibited the growth of cancer cells without affecting the normal cells [37]. Here, we found that Ocoxin exerted antitumor cytotoxicity and interfered with the cell cycle in three out of four tested melanoma cell lines. These results uncover the variability among cell types when dealing with the same treatment. Moreover, Ocoxin reduced the tumor area of BRAF-mutated melanoma metastasis in the lung in vivo. The antitumoral effect of Ocoxin is in part mediated by the modulation of the gene expression pattern of cancer cells. In this way, our previous studies reported that Ocoxin reverted the expression of several genes, some of which had been previously related to pancreatic carcinoma [9]. Regarding to melanoma, BRAF V600E mutation deregulates the MAPK signaling pathway that promotes cell proliferation and apoptosis evasion [1,4]. Interestingly, most of the genes downregulated by Ocoxin are related to proliferation and cell cycle regulation. Ocoxin also downregulates the MAPK signaling proteins implicated in cancer development, such as MAP3K9 and MAP4K3 [38,39,40,41]. In this regard, Xia et al. (2018) reported that MAP3K9 activated the MEK/ERK and NF-kB pathways, which promoted pancreatic cancer cell proliferation and the inhibition of apoptosis. The blockade of this kinase with a miR-7 resulted in the inhibition of these pathways, leading to a suppression of cancer cell proliferation and the induction of apoptosis [42]. Regarding to small cell lung carcinoma (NSCLC), the blockade of MAP4K3 inhibited tumor progression [41]. Moreover, Ocoxin downregulated the apoptotic inhibitor BIRC5, which was previously found to be upregulated in uveal and conjunctival melanoma [43,44]. Furthermore, the disruption of BIRC5 by CRISPR inhibited the progression of other malignancies, such as Acute Myelocytic Leukemia [45]. Therefore, it is tempting to hypothesize that Ocoxin may favor melanoma cell death through genetic modulation of the mentioned proteins.

As previously shown, the combination of Ocoxin and Vemurafenib increased the cytotoxicity of the BRAFi-targeted therapy by increasing the apoptosis of cancer cells in vitro. In line with these findings, Yang et al. (2017) reported that ascorbic acid or vitamin C, a component of Ocoxin, exhibited a potent cytotoxicity against melanoma cells and that it potentiated the effect of Vemurafenib [46]. Moreover, a sesquiterpene lactone plant derivative (DETD-35) had no effects on the wild-type MeWo and normal melanocytes but suppressed melanoma cell growth by increasing the apoptosis while overcoming Vemurafenib resistance in vivo [47]. This resistance is mainly facilitated by the components of the tumor microenvironment. It is known that the tumor microenvironment comprises predominantly CAFs, immune cells, and ECM, among other elements that interact closely with tumor cells. This crosstalk promotes tumor development and chemoresistance, thus offering potential therapeutic targets [48,49,50]. According to our results, TS-fibroblast secretomes increased the migration of the metastatic melanoma cells. This may be due to the epithelial to mesenchymal transition promoted by CAFs in tumor cells as reported previously [51]. Interestingly, Ocoxin reverted the promigratory effect produced by TS-fibroblasts in tumor cells, possibly partly due to Ocoxin cytotoxicity. The reported antitumor effect of Ocoxin may, in part, account for observed reduced migration. TS-fibroblast secretomes also increased the viability and resistance of cancer cells to Vemurafenib. As described by Hesler et al. (2016), the ECM protein CYR61 produced by CAFs induces chemoresistance to gemcitabine by downregulating nucleoside transporters [52], which may occur in this melanoma model. Even more, CAFs could activate MAPK and PI3K-AKT signaling pathways in cancer cells, promoting the resistance to RAF inhibitors by secreting HGF [28]. It is remarkable that Ocoxin sensitizes melanoma cells to targeted therapy by reducing the chemoresistance produced by TS-fibroblasts. Besides, Ocoxin reduces the secretion of proinflammatory mediators LGALS1, OPN, CCL5, and CCL9 chemokines by melanoma cells, which could result in a reduction of the recruitment and differentiation of mesenchymal stem cells into CAFs and decreased resistance to anticancer drugs of cancer cells [53,54]. Regarding to the modulation of the tumor microenvironment, our previous studies demonstrated that Ocoxin in vivo reduced the macrophage and hepatic stellate cell (HSC) migration into the liver metastatic foci of colon carcinoma [10,17], both known to play a central role on tumor progression [24,55]. In this regard, although Ocoxin alone reduced the metastatic development in the lung, it did not increase the effectiveness of the targeted therapy in vivo.

In summary, on the one hand, Ocoxin acts directly against tumor cells by reducing viability, increasing apoptosis, slowing down the cell cycle, and modifying the expression of protumoral genes. On the other hand, Ocoxin reduces fibroblast-mediated tumor progression by means of overcoming resistance against targeted therapy with the BRAF inhibitor and impairing fibroblast-mediated tumor cell migration. Thus, cotargeting both the tumor and TME elements may be effective strategy to deal with this malignancy [56,57]. Therefore, Ocoxin may constitute an effective coadjuvant agent for targeted therapy by directly acting upon metastasis tumor cells and by increasing BRAF inhibitors’ cytotoxicity while impairing tumor microenvironmental support.

## Figures and Tables

**Table 1:** Components and concentrations of Ocoxin nutritional complement mixture (Manufacturer’s product label).

Average Values (per 100 mL)	
Glycine	2.000 mg
Glucosamine	2.000 mg
Malic Acid	1.200 mg
Arginine	640 mg
Cysteine	204 mg
monoammonium glycyrrinate	200 mg
Ascorbic Acid	120 mg
Zinc Sulfate	80 mg
Green tea extract	25 mg
calcium pantothenate	12 mg
Piridoxine	4 mg
Manganese sulphate	4 mg
Cinnamon extract	3 mg
Sodium Benzoate	100 mg
Potasium Sorbate	100 mg
Maracuya Aroma	50 mg
Sucralose	24 mg

## Data Availability

Data is contained within the article or supplementary material.

## Supplementary Materials

The following are available online at https://www.mdpi.com/2072-6643/13/2/686/s1, Figure S1: In vivo treatment administration groups, Figure S2: The expression of MITF in YUMM 1.7, Figure S3: Apoptotic effect of the combination of Ocoxin and Vemurafenib in YUMM 1.7 melanoma cells, Figure S4: Apoptotic effect of the combination of Ocoxin and Vemurafenib in COLO-800 melanoma cells, Figure S5: Apoptotic effect of the combination of Ocoxin and Vemurafenib in HT-144 melanoma cells, Figure S6: Cytokine Antibody array membranes of control and Ocoxin treated cell secretomes, Figure S7: Percentage of mice with YUMM-1.7 cell lung metastasis.
