# Supplementary material for: Ocoxin Increases the Antitumor Effect of BRAF Inhibition and Reduces Cancer Associated Fibroblast-Mediated Chemoresistance and Protumoral Activity in Metastatic Melanoma
Source: Nutrients. 2021 Feb 21;13(2):686. doi: 10.3390/nu13020686 (PMC7924874; doi:10.3390/nu13020686)
Supplement: Supplementary file 1 [file nutrients-13-00686-s001.pdf]

nutrients

Supplemental Figures

Ocoxin Increases the Antitumor Effect of BRAF Inhibition and Reduces Cancer Associated Fibroblast-Mediated Chemo-resistance and Protumoral Activity in Metastatic Melanoma

Aitor Benedicto [1], Iera Hernández-Unzueta [1], Eduardo Sanz [2] and Joana Márquez [1*]

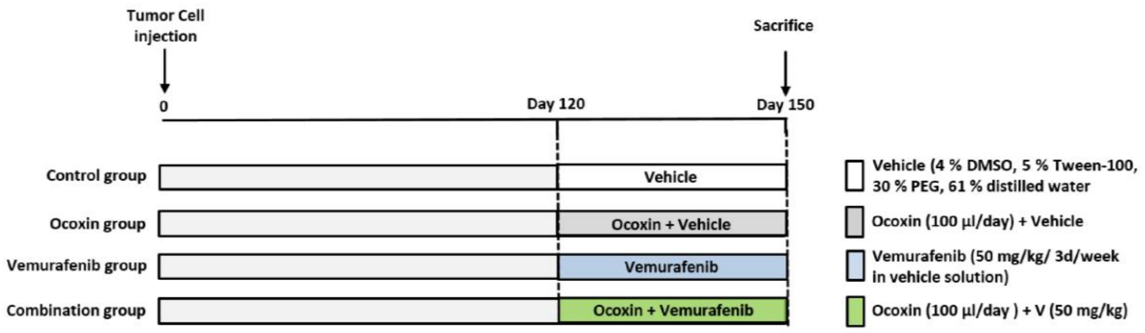

Figure S1. In vivo treatment administration groups.

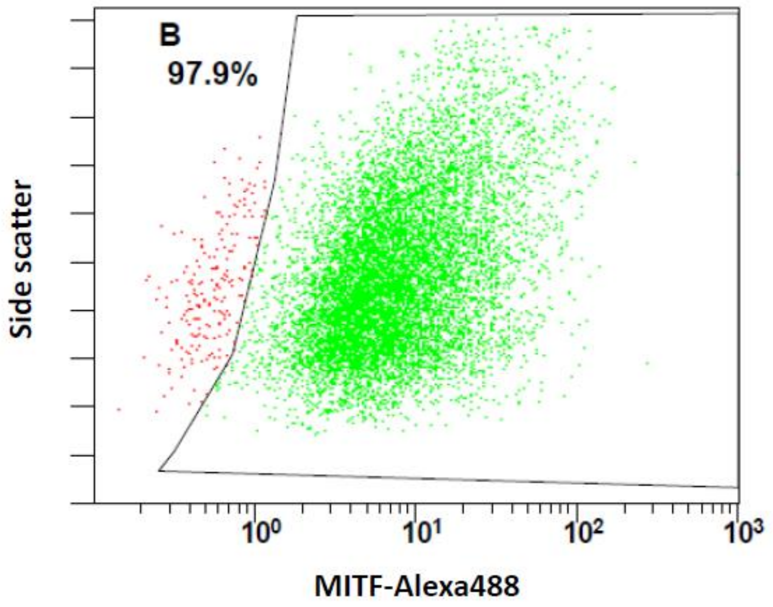

Figure S2. The expression of MITF in YUMM 1.7 melanoma cell line by Flow Citometry.

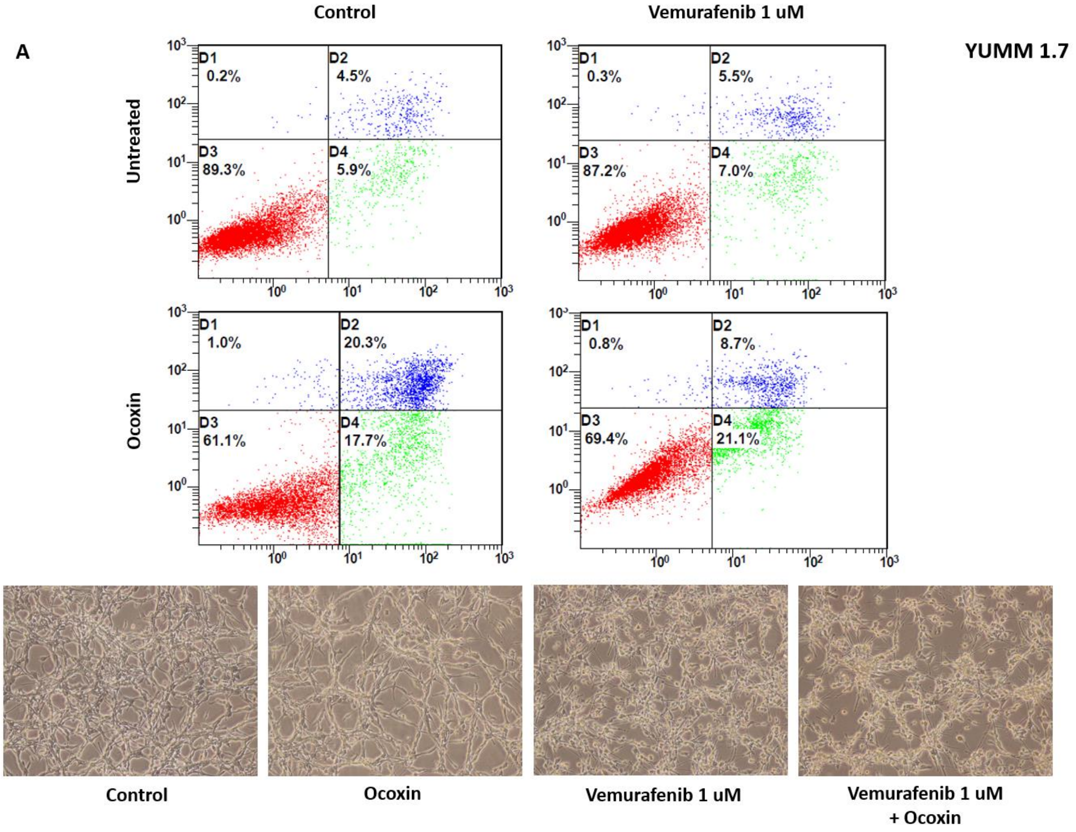

Figure S3. Apoptotic effect of the combination of Ocoxin and Vemurafenib in melanoma cells.

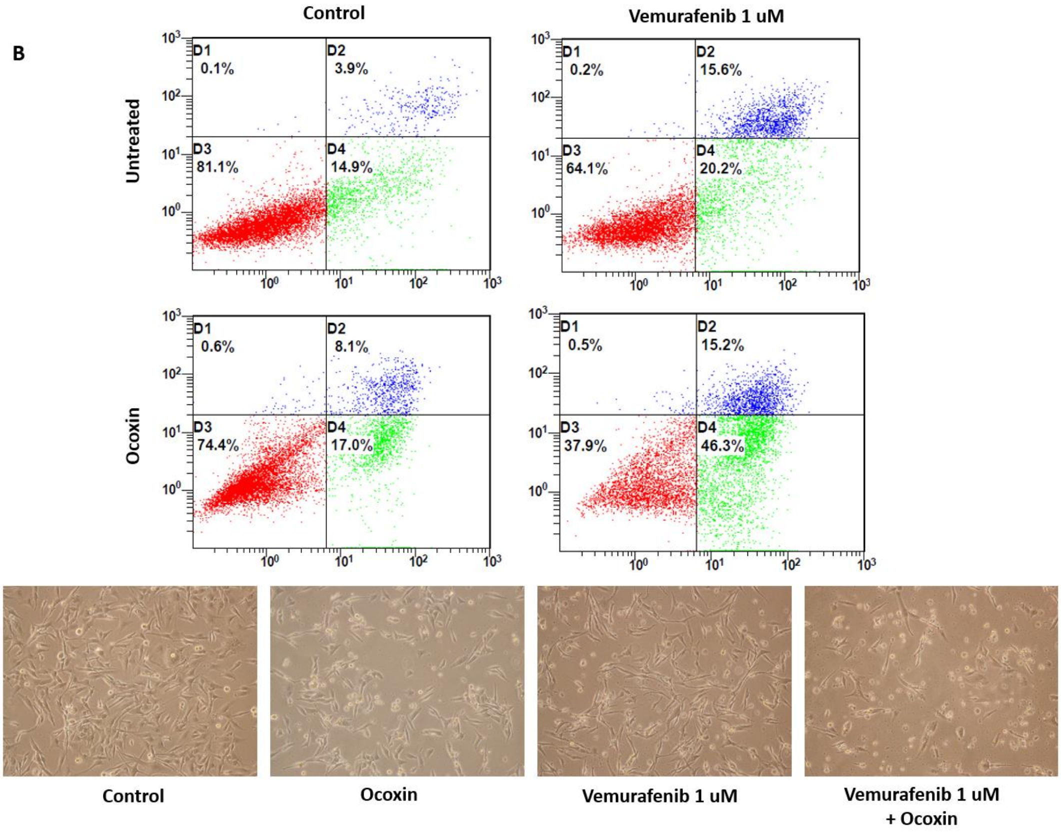

Figure S4. Apoptotic effect of the combination of Ocoxin and Vemurafenib in COLO-800 melanoma cells.

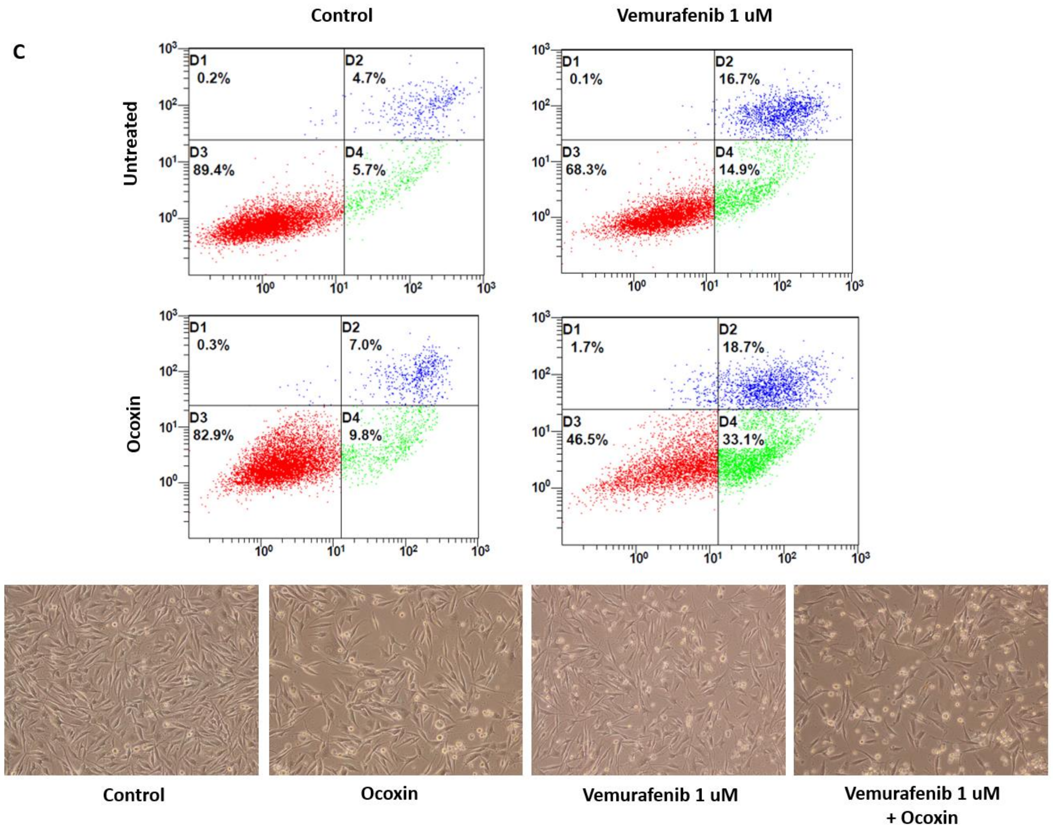

Figure S5. Apoptotic effect of the combination of Ocoxin and Vemurafenib in HT-144 melanoma cells.

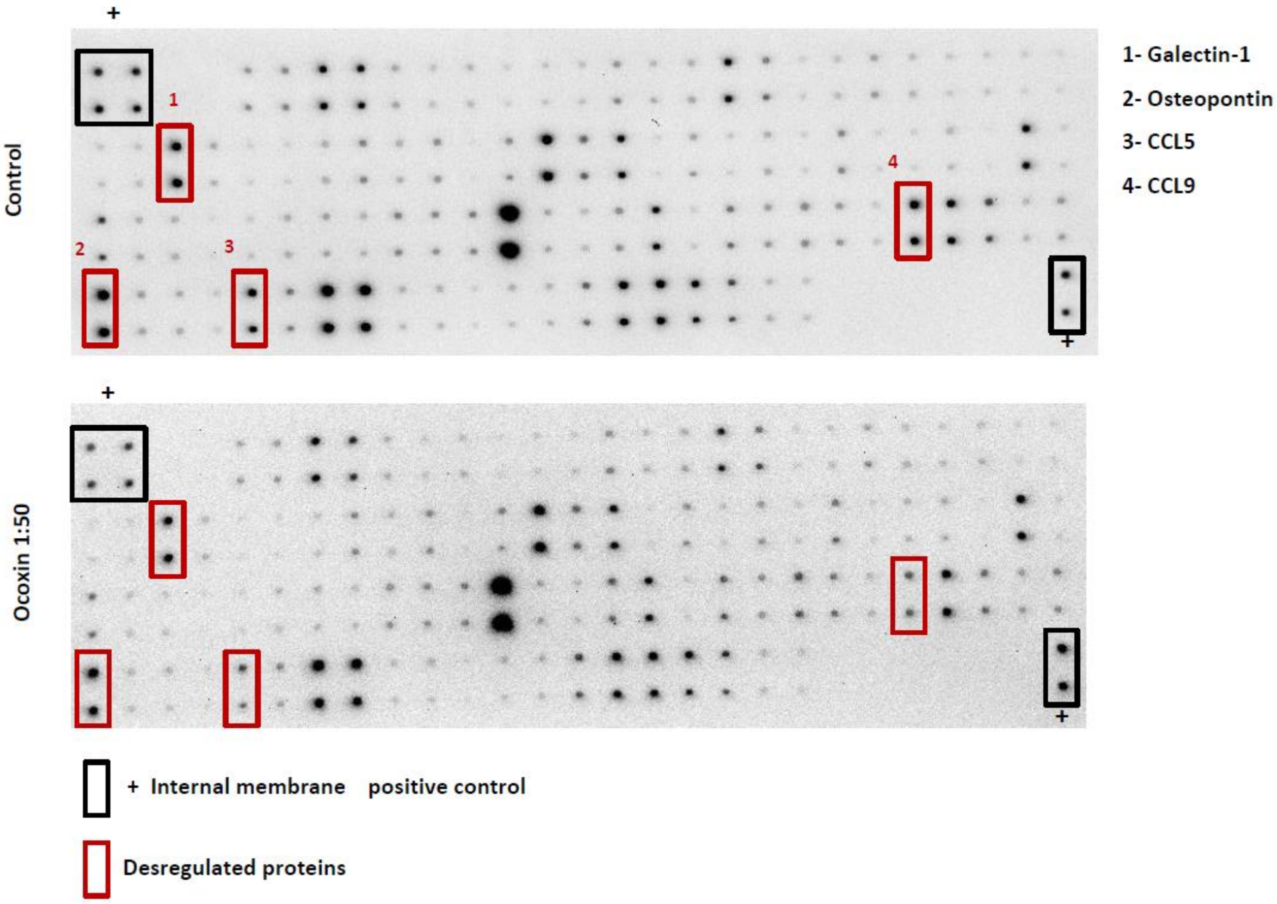

Figure S6. Cytokine Antibody array membranes of control and Ocoxin treated cell secretomes.

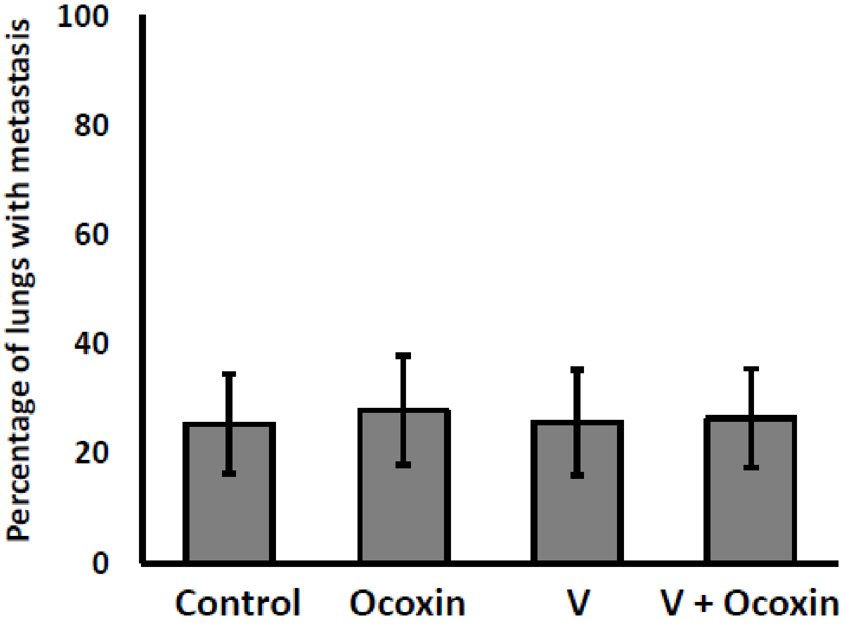

Figure S7. Percentage of mice with YUMM 1.7 cell lung metastasis.

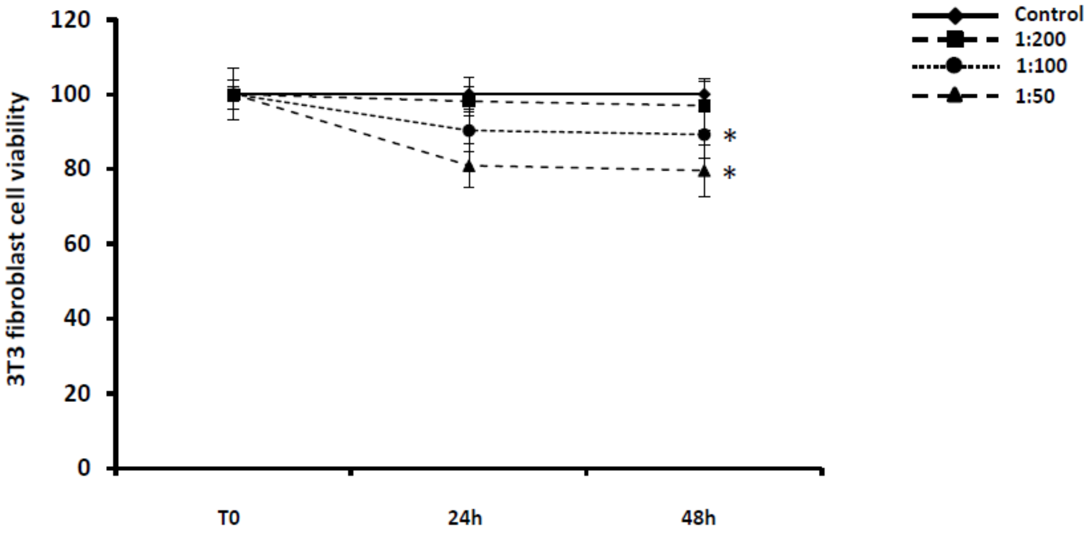

Figure S8. 3T3 fibroblast viability upon Ocoxin treatment.

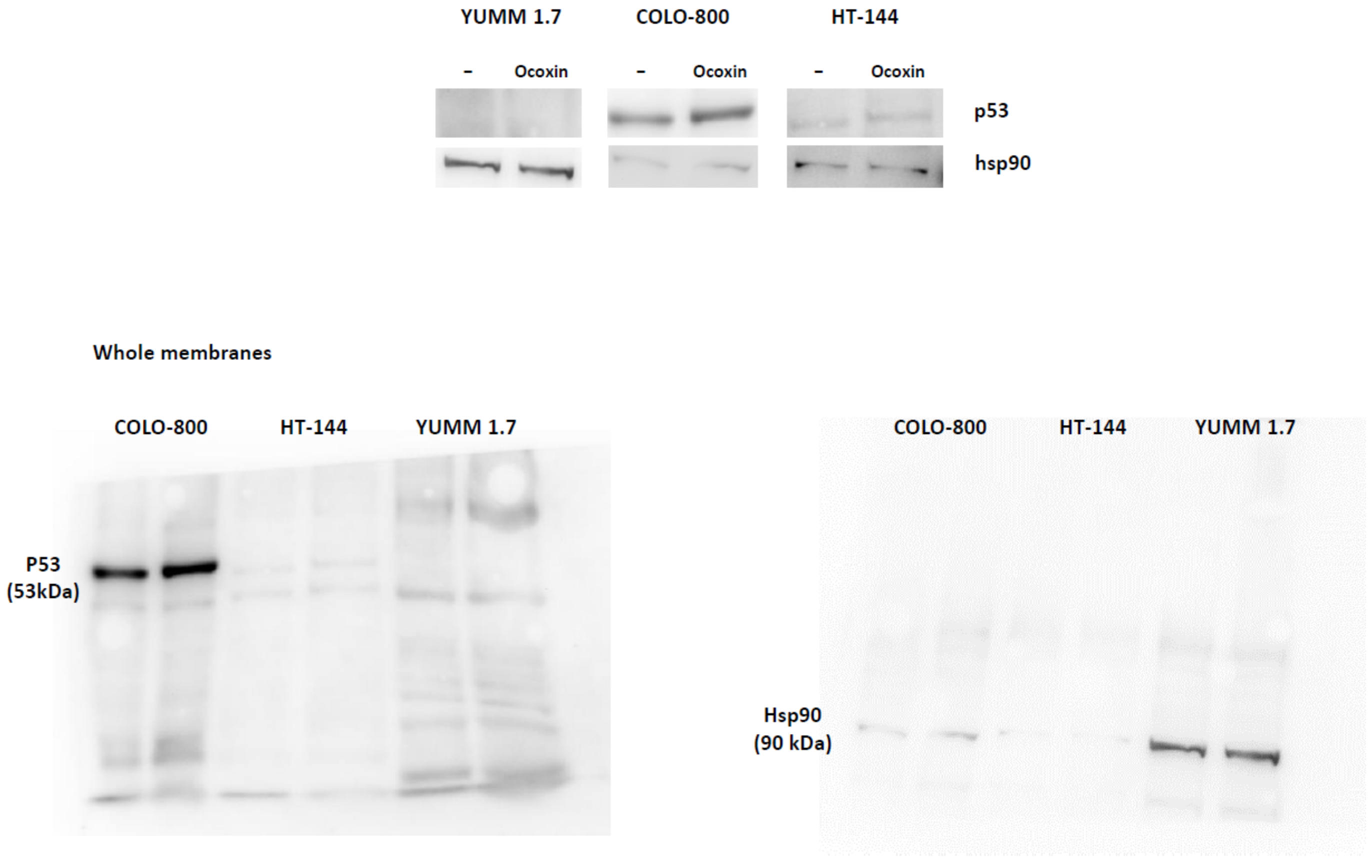

Figure S9. The western blot obtained for p53 (24 h).

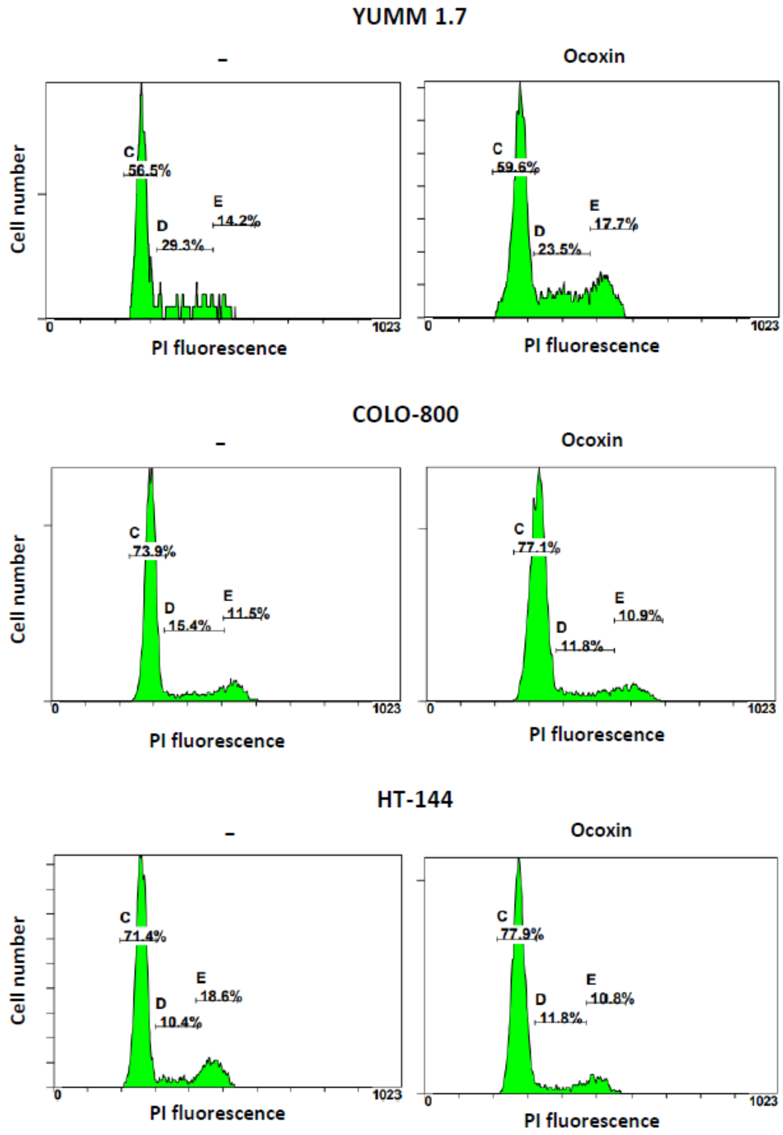

Figure S10. Cell cycle for 24 hours.
